# Lactic Acid Production from Acid Hydrolysate of *Ulva pertusa* as a Sustainable Biomass Feedstock

**DOI:** 10.3390/microorganisms14040788

**Published:** 2026-03-31

**Authors:** Yoojin Choi, Hyeongjin Hwang

**Affiliations:** Department of Chemical and Biological Engineering, Chungwoon University, 113, Sukgol-ro, Michuhol-gu, Incheon 22100, Republic of Korea; yjchoi216@chungwoon.ac.kr

**Keywords:** *Ulva pertusa*, lactic acid fermentation, *Lactobacillus*, seaweed hydrolysate, sustainable biomass, marine biorefinery

## Abstract

The development of sustainable alternatives to fossil-based feedstocks is a global priority in light of climate change and resource depletion. Seaweeds, particularly green seaweeds, represent promising candidates for biorefinery applications due to their rapid growth, high carbohydrate content, and non-competition with arable land. In this study, the feasibility of lactic acid production from acid hydrolysates of the green seaweed *Ulva pertusa* was systematically investigated. Proximate composition analysis revealed that dried *Ulva pertusa* contained 52.3% carbohydrates, highlighting its suitability as a fermentation substrate. Acid hydrolysis with dilute sulfuric acid released 23.8 g of fermentable monosaccharides per 100 g of biomass, with L-rhamnose and D-glucose as the predominant sugars. Fermentation experiments were conducted using five *Lactobacillus* strains (*L. casei*, *L. plantarum*, *L. brevis*, *L. salivarius*, and *L. rhamnosus*). Among these, *L. rhamnosus* and *L. salivarius* achieved the highest lactic acid yields (0.66 g g^−1^), followed by *L. plantarum* (0.63 g g^−1^), whereas *L. casei* and *L. brevis* exhibited comparatively lower yields (0.46 and 0.39 g g^−1^, respectively). Time-course analysis demonstrated that the superior strains reached maximum productivity within 9 h, significantly faster than typical lignocellulosic feedstocks such as corn stover, which require extensive pretreatment and longer fermentation times. Furthermore, the mineral-rich composition of *Ulva pertusa* (notably Mg^2+^ and Ca^2+^) provided intrinsic nutrients that supported microbial growth, thereby reducing the requirement for external supplementation. Comparative evaluation with lignocellulosic hydrolysates confirmed that *Ulva pertusa* offers higher efficiency, faster kinetics, and lower process complexity. To our knowledge, this work represents the first comprehensive assessment of multiple *Lactobacillus* strains for lactic acid production from *Ulva pertusa* hydrolysates. The findings highlight the unique advantages of green seaweeds as a sustainable biomass resource and contribute to the advancement of marine biomass-based biorefineries. Future studies should focus on improving the utilization of non-fermentable sugars, optimizing fermentation strategies, and evaluating techno-economic feasibility on an industrial scale.

## 1. Introduction

The transition from fossil-based resources to renewable biomass feedstocks has become an urgent global priority in response to climate change and the depletion of petroleum reserves [1]. Among various candidates, seaweed has emerged as promising raw material for biorefinery applications due to its rapid growth rates, high carbohydrate content, and cultivation that does not compete with food production on arable land [2]. In particular, red, green, and brown seaweeds such as *Gelidium*, *Ulva*, *Undaria* and *Laminaria* have attracted increasing attention as sustainable alternatives to conventional terrestrial crops [3,4]. Unlike lignocellulosic biomass, seaweeds are free of lignin, thereby avoiding the need for harsh physicochemical pretreatments, which are typically energy-intensive and costly [5]. This unique advantage not only reduces processing costs but also minimizes environmental impact, positioning seaweeds as an efficient and eco-friendly biomass source [6]. While seaweed offers clear advantages over lignocelluloses, its practical use also faces challenges such as seasonal variation in composition, high ash and salinity, high moisture content, and logistical constraints related to harvesting and storage. Lactic acid is one of the most versatile platform chemicals, with extensive applications across the food, pharmaceutical, textile, and leather industries [7]. Traditionally, lactic acid is produced via microbial fermentation using carbohydrate-rich feedstocks such as corn starch, sugarcane juice, and molasses [8]. However, the increasing global demand for lactic acid, particularly driven by its use as a monomer for polylactic acid (PLA), underscores the urgent need to diversify feedstocks [9]. PLA is a biodegradable, thermoplastic, and high-strength biopolymer that has attracted considerable interest as a sustainable alternative to petroleum-based plastics [10]. The rapid expansion of markets for biodegradable plastics further emphasizes the necessity of developing cost-effective and renewable routes for lactic acid production [11].

In recent years, efforts have been made to produce lactic acid from non-food renewable resources, including lignocellulosic biomass, food waste, and agricultural residues [12,13]. While these materials are abundant, their high lignin content and structural complexity require extensive pretreatment, which increases both costs and energy consumption [14]. Seaweeds, by contrast, can be cultivated in marine environments without freshwater or fertilizers and contain large amounts of easily hydrolyzable carbohydrates, such as glucose, rhamnose, and xylose [15]. In addition, seaweeds are rich in minerals, which may serve as natural nutrients for microbial growth, potentially reducing the need for supplementation during fermentation [16]. These characteristics make seaweeds a particularly attractive feedstock for microbial lactic acid production [17].

Previous studies have explored the utilization of various seaweeds to produce bioethanol, methane, and other value-added chemicals [18,19]. For example, *Gelidium amansii* has been studied as a bioenergy resource [20], and *Ulva lactuca* has been examined for its potential in methane generation and combustion [21]. Other macroalgae, including *Gracilaria corticata*, have been evaluated for lactic acid production using *Lactobacillus* strains [22]. However, systematic investigations of lactic acid production from green seaweeds remain relatively scarce. In particular, *Ulva pertusa*, which is widely distributed along the coast of Jeju Island in Korea, has not been extensively studied despite its abundance and favorable biochemical composition [23]. The high carbohydrate content of *Ulva pertusa* suggests that it could serve as a viable carbon source, while its mineral-rich composition may provide intrinsic growth-promoting factors for lactic acid bacteria [24].

The present study was therefore designed to evaluate the feasibility of producing lactic acid from *Ulva pertusa* hydrolysates. First, the proximate composition, elemental composition, and mineral content of dried *Ulva pertusa* biomass were analyzed to assess its biochemical suitability as a fermentation substrate. Acid hydrolysis using dilute sulfuric acid was then performed to release fermentable sugars, and the resulting hydrolysates were subjected to fermentation by five *Lactobacillus* strains (*L. casei*, *L. plantarum*, *L. brevis*, *L. salivarius*, and *L. rhamnosus*). Fermentation performance was evaluated in terms of sugar utilization, lactic acid yield, and by-product formation.

To our knowledge, few studies have systematically examined lactic acid production from *Ulva pertusa* hydrolysates. By directly comparing the efficiency of multiple *Lactobacillus* strains and benchmarking the fermentation results against lignocellulosic feedstocks such as corn stover [25], this work provides novel insights into the suitability of green seaweed as a sustainable and efficient raw material for lactic acid fermentation. The findings contribute to the broader development of seaweed-based biorefineries, which offer significant promise as environmentally friendly and economically competitive alternatives to traditional terrestrial biomass systems [25]. Although a few studies have reported lactic acid fermentation from *Ulva* or other seaweed hydrolysates, these investigations typically evaluated only single *Lactobacillus* strains or employed differing hydrolysis and culture conditions, hindering direct comparison. Here, we conduct the first systematic, side-by-side comparative evaluation of five *Lactobacillus* strains on a single *Ulva pertusa* hydrolysate under identical culture conditions, allowing quantitative assessment of strain-specific kinetics and selectivity.

In this study, we evaluate strain-specific performance on *Ulva pertusa* hydrolysates and identify fast, high-yielding candidates relative to conventional lignocellulosic benchmarks. Moreover, we characterized the dried biomass (i.e., proximate, elemental, and mineral composition), produced fermentable sugars via dilute-acid hydrolysis, and fermented the resulting hydrolysates as the sole carbohydrate source using five *Lactobacillus* strains, including *L. casei*, *L. plantarum*, *L. brevis*, *L. salivarius*, and *L. rhamnosus*. Finally, cell growth, residual sugars, and organic acids were quantified to calculate lactic acid yield (g g^−1^) and productivity (g L^−1^ h^−1^). This comparative approach offers practical insight into strain selection and process optimization in *Ulva pertusa*-based biorefineries.

## 2. Materials and Methods

### 2.1. Biomass Preparation and Proximate Composition

Fresh samples of *Ulva pertusa* were collected in March 2020, from the coast of Jeju Island, Korea. The biomass was thoroughly washed with tap water to remove salts, sand, and epiphytes, followed by drying at 60 °C until a constant weight was achieved. The dried biomass was ground into fine powder using a laboratory mill, suspended in distilled water, and then filtered through a 0.2 µm membrane to remove coarse residues and obtain a homogeneous feedstock for subsequent hydrolysis. Proximate composition analysis was performed according to the *Korea Food Code* [26]. Moisture content was determined by the air-oven drying method at 105 °C, while crude protein was estimated from total nitrogen content using the Kjeldahl method with a conversion factor of 6.25. Ash content was measured gravimetrically by incineration at 550 °C until constant weight, and crude lipid content was determined by ether extraction. The carbohydrate fraction was calculated by subtracting the sum of moisture, protein, ash, and lipid contents from 100%.

### 2.2. Elemental and Mineral Analyses

Elemental composition (C, H, O, N, S, and P) was determined using an elemental analyzer (EA 1108, ThermoFinnigan, Fisons Instrument Co., Milan, Italy) at 1000 °C, with WO_3_/Cu (nickel-plated carbon, nickel wool, soda lime, and magnesium perchlorate anhydrone for oxygen) as catalysts. For mineral analysis, 0.5 g of dried *Ulva pertusa* powder was digested using a hot-block system (DigiPREP HT 250, SCP Science, Quebec, QC, Canada) with a HNO_3_:H_2_O_2_ = 5:1 (*v*/*v*) acid mixture (8 mL HNO_3_ + 2 mL H_2_O_2_). The program comprised 15 min ambient soak, ramp to 120 °C (30 min), hold 120 °C for 90 min, then cool to room temperature. The digest was brought to a final volume of 50 mL with ultrapure water, filtered (0.22 µm), and analyzed by ICP-OES. Method blanks and matrix-spike samples were included for quality assurance. The digest was analyzed by Inductively Coupled Plasma–Optical Emission Spectrometry (ICP-OES; OPTIMA 7300DV, PerkinElmer, Waltham, MA, USA). Blank values were subtracted to correct background signals.

### 2.3. Acid Hydrolysis of Ulva pertusa Biomass

Dilute acid hydrolysis was carried out using 0.5 M H_2_SO_4_ (Sigma-Aldrich, St. Louis, MO, USA) as the catalyst. Dried *Ulva pertusa* powder (100 g) was suspended in 1 L of acid solution and autoclaved at 120 °C for 2 h. The hydrolysate was subsequently neutralized with CaCO_3_ (Junsei Chemical, Tokyo, Japan) until pH 6.5–7.0, followed by centrifugation at 8000 rpm for 15 min to remove precipitates. The supernatant was filtered through a 0.22 µm membrane filter and stored at 4 °C prior to fermentation. Released monosaccharides were analyzed using high-performance liquid chromatography (HPLC). Monosaccharides (L-rhamnose, D-glucose, D-xylose, D-glucuronic acid, and D-glucuronolactone) were quantified by HPLC (Agilent 1260 Infinity, Agilent Technologies, Santa Clara, CA, USA) equipped with a refractive index (RI) detector using an Aminex HPX-87H column (300 mm × 7.8 mm, Bio-Rad, Hercules, CA, USA) maintained at 35 °C. The mobile phase was 5 mM H_2_SO_4_ at a flow rate of 0.6 mL min^−1^, and the injection volume was 20 µL. Samples were centrifuged at 10,000× *g* for 5 min and filtered through 0.22 µm polytetrafluoroethylene filters prior to injection. Lactic, acetic, and succinic acids were determined at 210 nm by the same column, while furfural and 5-Hydroxymethylfurfural (5-HMF) were monitored at 280 nm.

### 2.4. Microorganisms and Culture Conditions

Five *Lactobacillus* strains were used in this study: *L. brevis* (KCTC 3498), *L. casei* (KCTC 3260), *L. plantarum* (KACC 11451), *L. rhamnosus* (KCTC 3237), and *L. salivarius* (KACC 10006). All strains were obtained from the Korean Collection for Type Cultures (KCTC) and Korean Agricultural Culture Collection (KACC). Pre-cultures were prepared in MRS-based medium (Difco Laboratories, Detroit, MI, USA) containing (per liter): 20 g glucose, 10 g peptone, 10 g beef extract, 5 g yeast extract, 2 g K_2_HPO_4_, 5 g sodium acetate, 2 g triammonium citrate, 0.2 g MgSO_4_·7H_2_O, 0.2 g MnSO_4_·4H_2_O, and 1 mL Tween 80. Pre-cultures were incubated at 30 °C for *L. casei* and *L. brevis* and at 37 °C for *L. plantarum*, *L. rhamnosus*, and *L. salivarius*.

### 2.5. Lactic Acid Fermentation

For fermentation experiments, the medium consisted of (per liter): 10 g peptone, 10 g beef extract, 5 g yeast extract, 2 g K_2_HPO_4_, 5 g sodium acetate, 2 g triammonium citrate, 0.2 g MgSO_4_·7H_2_O, 0.2 g MnSO_4_·4H_2_O, and 1 mL Tween 80. *Ulva pertusa* hydrolysate (1% *w*/*v*) served as the primary carbon source. Fermentations were conducted in 200 mL Erlenmeyer flasks with 100 mL working volume, incubated at 170 rpm on an orbital shaker under microaerobic, non-pH-controlled batch conditions for lactic acid bacteria. The initial total monosaccharide concentration in the *Ulva pertusa* hydrolysate was 10.8 g L^−1^, enabling direct comparison across all fermentation conditions. Cultures of *L. brevis* and *L. casei* were maintained at 30 °C, while *L. plantarum*, *L. rhamnosus*, and *L. salivarius* were incubated at 37 °C. Samples were periodically collected for analysis of cell growth, sugar consumption, and lactic acid production.

### 2.6. Analytical Methods

The concentrations of fermentation metabolites, including lactic acid and acetic acid, were determined using HPLC (ACME 9000, Younglin Instrument, Anyang, Republic of Korea) equipped with a UV–Vis detector (210 nm) and a refractive index (RI) detector. An Aminex HPX-87H column (Bio-Rad, Hercules, CA, USA) maintained at 35 °C was used with 5 mM H_2_SO_4_ as the mobile phase at a flow rate of 0.6 mL/min. The injection volume was 20 µL. Cell growth was monitored by measuring optical density at 600 nm (OD_600_) using a UV spectrophotometer (UV-1700, Shimadzu Corp., Kyoto, Japan).

### 2.7. Calculation of Lactic Acid Yield

The lactic acid yield (g g^−1^) was calculated according to Equation (1):

(1) $$Y_{LA/s}\left({g\;g}^{-1}\right)=\;\frac{C_{LA}-C_{LA,0}}{C_{S,0}-C_{S,t}}$$

where *C_LA_* and *C_LA_*_,0_ are the lactic acid concentrations (%, *w*/*w*) at a given time and at the initial time, respectively, and *C_S_*_,0_ and *C_S_*_,*t*_ represent the initial and residual sugar concentrations (%, *w*/*w*) at the corresponding time points.

## 3. Results and Discussion

### 3.1. Chemical Composition of Ulva pertusa

The biochemical composition of *Ulva pertusa* was analyzed to evaluate its suitability as a fermentation substrate. As shown in Table 1, the dried biomass contained 52.3% carbohydrates, which is higher than that reported for many terrestrial lignocellulosic biomasses such as corn stover (35–40%) [14,15]. This high carbohydrate content underscores the potential of *Ulva pertusa* as a sustainable carbon source for microbial fermentation.

Elemental analysis revealed that *Ulva pertusa* contained relatively high levels of sulfur compared to land-based plants. Sulfur plays a crucial role in microbial metabolism, serving as a component of amino acids, vitamins, and cofactors [17]. Also, a portion of sulfur is present in sulfated polysaccharides such as ulvan, which is characteristic of *Ulva* species. Additionally, the total mineral content of *Ulva pertusa* was 4.28%, with magnesium and calcium accounting for nearly 77% of the total mineral fraction. Both Mg^2+^ and Ca^2+^ are known cofactors for key glycolytic enzymes in lactic acid bacteria [18], which could enhance sugar metabolism and energy generation. From an industrial perspective, the presence of such intrinsic nutrients may reduce the need for external supplementation, an advantage compared with lignocellulosic biomass [19]. The composition of *Ulva pertusa* in this study is consistent with previous reports for green seaweeds, showing high levels of rhamnose and uronic acids typical of ulvan polysaccharides. Our values align with those reported for *Ulva* pertusa and *Ulva prolifera*, and fall within the expected range for Chlorophyta. Compared with brown (*Laminaria* spp.) and red (*Gracilaria* spp.) seaweeds, *Ulva pertusa* contains lower glucose but higher mineral ash and uronate fractions, reflecting its characteristic ulvan-rich cell wall composition.

### 3.2. Acid Hydrolysis and Sugar Composition

Dilute acid hydrolysis of dried *Ulva pertusa* (100 g) yielded 23.8 g of monosaccharides per 100 g dry biomass. The hydrolysate was mainly composed of L-rhamnose, followed by D-glucose, D-glucuronic acid, D-xylose, and D-glucuronolactone (Table 1).

The predominance of L-rhamnose is notable, as this sugar originates primarily from ulvan, a sulfated polysaccharide abundant in green seaweeds [13,20]. Unlike lignocellulosic biomasses, which mainly contain glucose and xylose [14,15], the presence of rhamnose provides a unique advantage since several *Lactobacillus* species, such as *L. plantarum* and *L. rhamnosus*, are able to metabolize this sugar [21]. The availability of both rhamnose and glucose likely contributed to the higher lactic acid yields observed in subsequent fermentations.

However, the hydrolysate also contained uronic acids (glucuronic acid and glucuronolactone), which are not readily metabolized by most conventional lactic acid bacteria [22]. This limitation suggests that part of the carbohydrate fraction remains unutilized, thereby constraining the theoretical maximum lactic acid yield. Future strategies could include metabolic engineering to expand the substrate spectrum of *Lactobacillus* strains [23] or the use of microbial consortia capable of degrading uronic acids [24].

### 3.3. Lactic Acid Fermentation by Lactobacillus Strains

Five *Lactobacillus* strains were tested for lactic acid fermentation using *Ulva pertusa* hydrolysates as the sole carbon source. The results are summarized in Table 2. The highest lactic acid yields were obtained with *L. rhamnosus* and *L. salivarius* (0.66 g g^−1^), followed closely by *L. plantarum* (0.63 g g^−1^). These strains (Group A) exhibited efficient sugar utilization and rapid fermentation kinetics. In contrast, *L. casei* and *L. brevis* (Group B) displayed lower lactic acid yields (0.46 and 0.39 g g^−1^, respectively).

The enhanced performance of Group A strains may be attributed to their metabolic versatility, enabling them to co-utilize rhamnose and glucose [21,25]. Previous reports demonstrated that *L. rhamnosus* and *L. plantarum* are robust fermenters capable of efficient lactic acid production from mixed sugar substrates, including those derived from seaweed hydrolysates [9,14]. These findings suggest that certain *Lactobacillus* strains are better adapted to the unique sugar profile of *Ulva pertusa* compared with other strains.

### 3.4. Fermentation Kinetics and Metabolic Shifts

**Figure 1:**
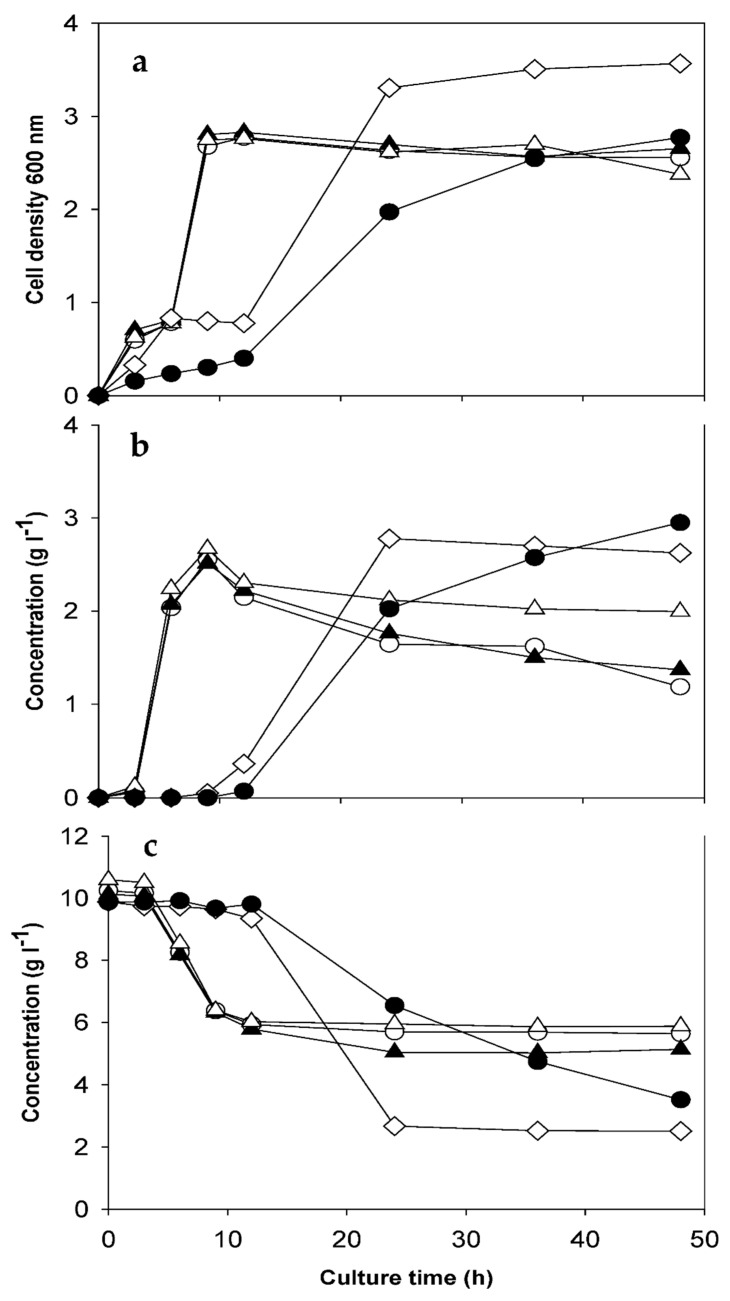
Time-course profiles of lactic acid fermentation of *Ulva pertusa* hydrolysate by different *Lactobacillus* strains. (**a**) Cell density (OD_600_), (**b**) lactic acid production, and (**c**) total sugar consumption. ●, *L. casei*; ◇, *L. brevis*; △, *L. plantarum*; ▲, *L. salivarius*; ○, *L. rhamnosus*.

The time-course profiles of fermentation are presented in Figure 1. Group A strains reached maximum lactic acid concentrations (2.5–2.7 g L^−1^) and cell densities (1.8–2.0 × 10^8^ CFU mL^−1^) within 9 h, indicating rapid utilization of available sugars. Their lactic acid productivities averaged 0.28–0.30 g L^−1^ h^−1^, with yields of 0.63–0.66 g g^−1^. After reaching peak production, lactic acid concentrations declined, suggesting its conversion into acetic acid through secondary metabolic pathways under nutrient limitation and low-pH stress [15].

In contrast, Group B strains (*L. brevis* and *L. casei*) required 24–48 h to reach maximum lactic acid production titers of 2.8–3.0 g L^−1^, demonstrating slower fermentation kinetics. Their total sugar consumption was higher (6.5–7.4 g L^−1^) compared with Group A (3.9–4.4 g L^−1^), but yields were lower (0.39–0.46 g g^−1^) due to carbon diversion into by-products, primarily acetic acid (1.5–1.9 g L^−1^), 1,2-propanediol (0.6 g L^−1^), and acetaldehyde (0.2–0.5 g L^−1^). Their consumption profiles indicated a diauxic growth pattern, in which microorganisms preferentially metabolized glucose and rhamnose before shifting to xylose and uronic acids [14,22]. Such sequential sugar utilization has been reported in lactic acid bacteria and can limit productivity. Also, these results showed that the heterofermentative metabolism (e.g., *L. brevis* and *L. casei*), which divert part of the carbon flux from sugars toward by-products such as acetic acid, ethanol, and CO_2_. As a result, their lactic acid yields remained lower despite higher total sugar consumption, consistent with previously reported metabolic characteristics of these species.

The faster kinetics and higher yields of Group A strains underline their industrial potential, as a shorter fermentation time (e.g., 9 h vs. 24–48 h) directly enhances process efficiency and cost-effectiveness [5,6].

The lactic acid titers and yields obtained in this study are comparable to those reported in the numerical study [22], which modeled lactic acid production from *Ulva prolifera* hydrolysates under similar sugar compositions. Our experimental yields (0.39–0.66 g g^−1^) fall within the predicted range and are also consistent with other seaweed fermentation studies using *Enteromorpha* and *Gracilaria* hydrolysates (0.35–0.70 g g^−1^). This understanding supports the robustness of *Ulva pertusa* hydrolysates as a viable feedstock for lactic acid fermentation.

### 3.5. Comparison with Lignocellulosic Feedstocks

To benchmark the performance of *Ulva pertusa*, fermentation results were compared with those obtained using lignocellulosic feedstocks. Cui et al. reported lactic acid yields of 0.59 g g^−1^ for *L. rhamnosus* and 0.54 g g^−1^ for *L. brevis* when fermenting alkali-pretreated corn stover hydrolysates [25]. In comparison, *Ulva pertusa* hydrolysates supported yields up to 0.66 g g^−1^, exceeding those obtained from corn stover.

This result is significant for two reasons. First, unlike lignocellulosic biomass, seaweeds lack lignin, thereby eliminating the requirement for harsh pretreatment processes [8,14]. Second, the mineral-rich composition of *Ulva pertusa* provides essential nutrients, which can partly replace external supplementation during fermentation [9,18]. These advantages enhance the economic and environmental feasibility of marine biomass as a sustainable feedstock for lactic acid production.

## 4. Conclusions

This study demonstrated the feasibility of producing lactic acid from *Ulva pertusa* hydrolysates using five different *Lactobacillus* strains. Among them, *L. rhamnosus* and *L. salivarius* achieved the highest lactic acid yields (0.66 g g^−1^) with rapid fermentation kinetics, outperforming conventional lignocellulosic feedstocks such as corn stover. The high carbohydrate content (52.3%) and intrinsic mineral composition of *Ulva pertusa* further highlight its advantages as a sustainable substrate, reducing the need for energy-intensive pretreatments and external nutrient supplementation. These findings underline the potential of *Ulva pertusa* as a promising marine biomass feedstock for lactic acid fermentation and contribute to the development of environmentally friendly and economically viable biorefinery processes. In future work, efforts should focus on enhancing the utilization of uronic acids, mitigating product inhibition, and evaluating the scale-up feasibility for industrial applications.

## Figures and Tables

**Table 1:** Proximate, Elemental, Mineral, and Hydrolysate Sugar Composition of *Ulva pertusa*.

Proximate Composition (g/100 g Dry Weight)		Ultimate Analysis (g/100 g Dry Weight)		Mineral Analysis (μg g^−1^ Dry Weight)		Sugar Composition of Hydrolysate (g/100 g Dry Weight)	
Carbohydrate	52.3	C	34.87	Mg	19,580	L-rhamnose	11.6
Protein	25.1	H	5.28	Ca	13,320	D-glucose	5.0
Lipid	0.1	O	46.54	K	3070	D-glucuronic acid	2.7
Ash	22.5	N	3.77	Fe	2980	D-glucuronolactone	2.2
		S	3.13	Al	2670	D-xylose	2.3
		P	0.12	Na	1060		
		others	6.29	Sr	120		
				Mn	50		
				Cu	20		
Total	100		100		42,750		23.8

**Table 2:** Lactic acid production and sugar utilization from *Ulva pertusa* hydrolysates by *Lactobacillus* strains.

Parameter	Group A			Group B	
	*L. rhamnosus*	*L. salivarius*	*L. plantarum*	*L. casei*	*L. brevis*
Cell density (OD_600_)	2.68	2.81	2.75	2.77	3.31
Total products (g L^−1^) *	5.2	4.9	5.3	6.7	6.8
Lactic acid (g L^−1^) *	2.6	2.5	2.7	3.0	2.8
Acetic acid (g L^−1^)	0.6	0.6	0.6	1.9	1.5
Succinic acid	1.8	1.7	1.8	1.0	1.4
1,2-propanediol	0.2	0.1	0.2	0.6	0.6
Acetaldehyde	-	-	-	0.2	0.5
L-rhamnose	2.2	2.4	2.5	3.0	3.2
D-glucose	1.4	1.3	1.4	1.4	1.4
D-xylose	0.3	0.1	0.3	0.3	1.4
D-glucuronic acid	0.0	0.0	0.0	1.7	1.3
D-glucuronolactone	0.1	0.1	0.2	0.2	0.2
Lactic acid yield (g g^−1^)	0.66	0.66	0.63	0.46	0.39

* Maximum lactic acid production occurred at fermentation time of 9 h for *L. rhamnosus*, *L. salivarius*, and *L. plantarum*, 24 h for *L. brevis*, and 48 h for *L. casei*, respectively.

## Data Availability

The original contributions presented in the study are included in the article, further inquiries can be directed to the corresponding author.
